# Dynamic Tracking and Real-Time Fall Detection Based on Intelligent Image Analysis with Convolutional Neural Network

**DOI:** 10.3390/s24237448

**Published:** 2024-11-22

**Authors:** Ching-Bang Yao, Cheng-Tai Lu

**Affiliations:** Department of Information Management, Chinese Culture University, Taipei 11114, Taiwan; yqb@faculty.pccu.edu.tw

**Keywords:** smart home care, fall posture analysis, facial recognition, real-time tracking, OpenPose

## Abstract

As many countries face rapid population aging, the supply of manpower for caregiving falls far short of the increasing demand for care. Therefore, if the care system can continuously recognize and track the care recipient and, at the first sign of a fall, promptly analyze the image to accurately assess the circumstances of the fall, it would be highly critical. This study integrates the mobility of drones in conjunction with the Dlib HOG algorithm and intelligent fall posture analysis, aiming to achieve real-time tracking of care recipients. Additionally, the study improves and enhances the real-time multi-person action analysis feature of OpenPose to enhance its analytical capabilities for various fall scenarios, enabling accurate analysis of the approximate real-time situation when a care recipient falls. In the experimental results, the system’s identification accuracy for four fall directions is higher than that of Google Teachable Machine’s Pose Project training model. Particularly, there is the significant improvement in identifying backward falls, with the identification accuracy increasing from 70.35% to 95%. Furthermore, the identification accuracy for forward and leftward falls also increases by nearly 14%. Therefore, the experimental results demonstrate that the improved identification accuracy for the four fall directions in different scenarios exceeds 95%.

## 1. Introduction

In traditional society, families have traditionally been the sole caregiving resource. In Taiwan, with the initiation of the “Senior Citizen Welfare Act” on 26 January 1980, the formalization of elderly welfare legislation began, bringing attention to the issue of caring for the elderly. In the past, elderly care was primarily carried out through manual labor. However, with the economic rise of Taiwan in the 1990s, there was a substantial demand for domestic labor, leading to a shortage of manpower for elderly care. Consequently, foreign labor was introduced to compensate for this shortage [1].

In recent years, surveillance devices have become ubiquitous and visible on streets, campuses, public facilities, and more. The application of surveillance devices in home care for the elderly is also a noteworthy topic [2,3]. However, the use of surveillance devices comes with the drawback of blind spots in the field of vision [4,5]. Therefore, this study explores how unmanned aerial vehicles (drones) can be employed to replace surveillance devices in caring for the elderly. The aim is to realize a vision of combining artificial intelligence with machinery to compensate for the shortage of manpower.

As countries around the world enter an “aging society”, falls have become one of the most common issues among the elderly. According to the World Health Organization (WHO) fact sheet, falls are the second leading cause of unintentional injury deaths globally, with the highest number of fatalities occurring among adults aged 60 and above [6]. The injuries caused by falls can range from minor soft tissue damage to life-threatening injuries [7], and different fall directions may result in varying levels of injury severity [8,9]. This increases the likelihood of severe injuries, placing a significant burden on society and families [10].

Elderly individuals may lose consciousness after falling and roll unconsciously, making it difficult for medical personnel to accurately identify the injured areas upon arrival. This can lead to a time-consuming and challenging process, ultimately increasing the risk of delayed treatment for the elderly. Additionally, older adults may struggle to recall the details of their fall, and sometimes the injured areas may not show obvious external injuries, making it difficult for doctors to determine which areas need to be examined by X-rays. Without an accurate fall record, doctors may be unable to pinpoint the optimal areas for examination, leading to delays in diagnosis and treatment, as well as unnecessary guesswork regarding the fall, which further increases the patient’s health risks.

Moreover, the demand for long-term care is rapidly increasing due to the aging society, while the declining birth rate and rising female labor force participation have shifted long-term care services from being primarily provided by families to being outsourced. This includes placing individuals in care institutions or nursing homes, or hiring caregivers to provide in-home care. However, whether through institutions or caregivers, this shift imposes a considerable financial burden on families.

Past related research on fall detection in the elderly primarily employed image recognition coupled with traditional surveillance cameras in experiments [2]. The advantages of this approach include the ability to recognize specific body postures and objects, as well as obtaining images in a timely manner. This significantly addresses the shortcomings of manual monitoring. However, the drawback of using traditional surveillance cameras is the tendency to have blind spots, making it challenging to recognize the posture of the care recipient at all times.

In recent years, some studies have recognized the limitations of traditional surveillance cameras in experimental setups. Consequently, they have opted for wearable sensors. Unlike traditional surveillance cameras, which may have blind spots and require confining the experimental subject to monitored spaces for detection, wearable sensors can be worn by the care recipient [11]. This allows continuous detection of the care recipient’s posture, reducing the limitations associated with blind spots [12]. The success rate of recognition with wearable sensors is also relatively higher than that of traditional monitoring devices [13,14].

However, wearable sensors have their drawbacks, as they require cooperation from the care recipient for their use. Therefore, they cannot be used for individuals with cognitive impairments or those who cannot cooperate. Additionally, wearable sensors are susceptible to the influence of parameters. Since wearable sensors do not use image recognition technology, they can be influenced into incorrect posture judgments, leading to a decrease in recognition accuracy.

In order to address the three major issues of the elderly not receiving timely treatment if they fall, the inability of traditional surveillance cameras to follow the care recipient, and the requirement for user cooperation with wearable sensors, this study combines the advantages of smart unmanned aerial vehicles (UAVs) that can autonomously track the care recipient and monitor without blind spots. It integrates facial recognition technology from artificial intelligence image recognition and analysis techniques. Additionally, we have enhanced the posture recognition technology using the OpenPose algorithm for identifying human body postures.

We utilize a human body posture coordinate algorithm and posture classification, resulting in a more refined human body posture recognition algorithm. This not only enables the identification of subtle body postures but also significantly improves the success rate of detailed human body posture recognition. With this approach, the care recipient can be tracked and monitored, allowing for the detection of any abnormal safety conditions. Through real-time information recognition technology and instant fall detection records, the latest monitoring images, fall timestamps, and the severity of falls are communicated to the family and medical personnel of the care recipient. This ensures that the care recipient does not miss the opportunity for timely medical intervention.

The smart UAV, coupled with artificial intelligence for real-time tracking and monitoring of the care recipient’s fall posture, not only addresses the shortcomings of traditional surveillance cameras but also eliminates the need for user cooperation with wearables. It can transmit the care recipient’s information back to the monitoring host without requiring the care recipient to wear any devices, providing optimal real-time information.

In summary, this study proposes a system for intelligent dynamic tracking and fall detection using deep learning methods. The system begins with a drone remote control module utilizing OpenCV to capture images taken by the drone. Subsequently, a face detection module employing Dlib HOG algorithm detects the face of the care recipient, enabling the drone to follow the recipient in real time. Further, a pose recognition module utilizes OpenPose to identify and mark joints of the human body, along with the defined Part Affinity Field (PAF), to find corresponding pose vectors. This allows the images captured by the drone to provide relevant information about the care recipient’s body.

However, although OpenPose’s PAF can effectively identify various joints of the human body, when certain body postures result in joint occlusion, there may be misinterpretation of the body posture. To address this issue, this study proposes the “Intelligent Fall Posture Analysis Algorithm”, which further analyzes different types of postures by using the coordinates of the joints and their relative heights and positions. This aims to effectively establish a posture classification model, thereby achieving real-time and highly accurate posture recognition.

Therefore, the intelligent fall posture analysis module developed in this study determines whether the care recipient is in a falling posture and provides a more detailed analysis of the type and direction of the fall, enabling the drone to detect falls effectively. The fall care module, using predefined conditions in Python libraries, assesses the severity of the fall and records the time of occurrence. The monitoring system transmits the recorded fall data—including the direction, severity, timestamp, and real-time footage of the fall—to the care recipient’s family and medical personnel.

This fall detection information is valuable for doctors when performing X-ray examinations. By providing real-time detection and recording of the fall posture, doctors can more accurately identify which areas of the care recipient require X-ray or even Computed Tomography examination. Additionally, based on the system’s real-time image analysis, doctors can assess potential causes of the fall, such as leg weakness, balance disorders, or accidental collisions. This information enables doctors to make more precise and timely diagnoses and treatments, helping the care recipient receive immediate and effective medical attention.

Furthermore, having this system accompany the care recipient alleviates concerns and stress for their family, knowing that the system can monitor and follow the recipient in real time. By utilizing drones and artificial intelligence to monitor body posture, the system developed in this research ensures both real-time tracking and fall detection of the care recipient. This framework forms the basis of the study presented in this paper.

The structure of this thesis is as follows: Section 1 is the introduction. Section 2 introduces the relevant literature and technologies, smart real-time home care, deep learning, fall detection applications, OpenPose, and Teachable Machine. Section 3 describes the system architecture and modules, explaining the five modules of the smart drone following and fall detection systems and their operational logic. Section 4 presents the experimental results, discussing experimental design and data analysis, as well as the accuracy and error sources in facial recognition and detection of falls in four directions. Section 5 discusses the proposed intelligent following and fall detection system, focusing on the analysis of experimental results, improvements to OpenPose, and a comparison between this system and Teachable Machine. Finally, Section 6 provides the conclusion and intentions for future work, summarizing the advantages and limitations of this system in recognizing human postures in four different fall directions, practical constraints in its application, and potential future development directions.

## 2. Materials and Methods

### 2.1. Smart Real-Time Home Care

In a global context, falling is a significant public health issue. According to the World Health Organization’s (WHO) manual on falls, an estimated 684,000 fatal falls occur each year worldwide, with over 80% taking place in low- and middle-income countries. Among these, individuals aged 60 and above experience the highest mortality due to falls. While not all falls result in fatalities, approximately 37.3 million people require medical care each year due to severe falls. Furthermore, falls contribute to over 38 million Disability-Adjusted Life Years (DALYs) lost annually [15], surpassing the combined impact of traffic injuries, drowning, burns, and poisoning in terms of years lived with disability [6].

When an elderly member of a household experiences a fall and sustains injuries, there arises a long-term need for care. Families address this need in various ways, with some opting for the presence of family members or caregivers to provide support directly, while others choose to entrust the elderly to care facilities designed for seniors. These care facilities concentrate on providing care for each elderly individual entrusted to them, utilizing a combination of human resources and surveillance devices [16]. However, the drawback of traditional surveillance devices lies in their blind spots, limiting the comprehensive care available to the elderly.

In recent years, technological advancements have led many care centers to complement traditional surveillance systems with wearable devices [17], creating a synergistic approach that results in intelligent real-time care [18]. This significantly improves upon the limitations of conventional surveillance devices. However, some families may find the cost of care centers prohibitive, or the increasing aging population might strain the capacity of such facilities. Consequently, home-based care has emerged as an alternative [19]. Home-based care is viewed as an extension or substitution for traditional hospitalization, shortening the duration of patient stays while ensuring continuity in medical care, thereby providing comprehensive support.

Previous research has explored the integration of various devices (such as surveillance systems [20], wearable devices [21], intelligent monitoring robots [22], etc.) with artificial intelligence to achieve the goal of smart home-based care [23]. Therefore, this study developed a drone with real-time recognition and tracking capabilities, integrating an intelligent real-time fall detection and analysis algorithm to implement a smart home care service for monitoring and notifications.

### 2.2. Deep Learning

Deep learning is a branch of machine learning that differs in its approach from traditional machine learning. While machine learning involves computers using algorithms to infer patterns and features from a vast amount of historical data provided by humans, deep learning draws inspiration from the functioning of the human brain’s neural networks. It employs multi-layered artificial neural networks to learn from extensive datasets.

Within various deep learning techniques, Convolutional Neural Networks (CNN) demonstrate outstanding performance in image recognition. The primary structure of CNN includes convolutional layers, pooling layers, and a final dense layer which is a fully connected layer. When progressing to the dense layer, CNN goes through multiple sets primarily responsible for feature extraction through convolutional and pooling layers [24].

The convolutional layer traverses the image using convolutional kernels to determine which features to enhance or weaken. The pooling layer selects key features from the output of the convolutional layer, dividing the feature map data and extracting the maximum values. After several sets of convolutional and pooling layers, the extracted features from the pooling layer are fed into the dense layer to calculate the predicted classification result.

### 2.3. The Application of Deep Learning in Fall Detection

In the previous literature, methods employed for fall detection primarily fall into the categories of wearable devices, environmental sensing devices, and computer vision-based fall detection [25]. The following will provide a literature review and discussion on each of these fall detection methods.

The part related to fall detection in wearable devices primarily involves using heart rate sensors [26], gyroscope sensors [27], or accelerometers to determine values and features indicative of a fall [28]. By connecting the detector to a system and employing machine learning, wearable devices for fall detection achieve higher accuracy and real-time transmission of sensor results. However, challenges arise when the wearable device is not worn by the user, leading to the inability to detect falls. Additionally, malfunctioning sensors can pose obstacles to fall detection. The use of wearable devices for fall detection also introduces discomfort for users due to the necessity of wearing them. Lastly, since wearable devices do not utilize visual methods for fall detection, there is a risk of being deceived by the parameters being sensed.

When used for fall detection, environmental sensing devices primarily employ methods such as detecting floor vibrations and sounds or utilizing ultrasonic and radar devices to sense features and numerical values indicative of a fall [29,30,31,32]. Like wearable devices, these sensing devices are connected to a system and often coupled with machine learning or neural networks for detection. Although environmental sensing devices generally exhibit slightly lower accuracy in fall detection compared to wearable devices, they offer real-time transmission of sensing results.

However, environmental sensing devices are susceptible to interference from environmental factors, and their setup costs are higher. Additionally, due to their limited sensing range, deploying a sufficient quantity to cover the entire daily living environment for elderly care may be necessary. Even after deployment, there is a possibility of blind spots in the sensor coverage.

In summary, this study employs unmanned aerial vehicles (UAVs) combined with facial detection and OpenPose body pose estimation for computer vision-based fall detection. In comparison to past research utilizing surveillance devices for computer vision-based fall detection, which can be susceptible to environmental factors such as obstructions or overlapping of individuals, resulting in the inability to detect falls, traditional surveillance cameras are fixed in position, making it challenging to cover the entire daily living environment. This limitation can lead to blind spots in practical applications.

Therefore, this study utilizes UAVs for real-time monitoring, low construction costs, and the ability to navigate obstacles during flight. Facial detection is employed to serve as a reference point for UAV system operation, addressing the drawbacks of traditional surveillance devices with potential blind spots. Furthermore, to mitigate the impact of environmental factors, this research utilizes OpenPose, allowing accurate prediction of obscured body joint points even in cases of overlapping individuals or obstruction by objects. This approach aims to enhance fall detection accuracy in challenging environmental conditions.

### 2.4. OpenPose

OpenPose, developed by researchers at Carnegie Mellon University (CMU), is an open-source model that is considered one of the best real-time human pose estimation methods currently available. In a study conducted by S. Xiong et al., the researchers utilized the RULA/REBA human posture assessment methods for measurement. The article mentioned that OpenPose outperformed Kinect in measuring joint angles and conducting semi-automatic human posture assessments. OpenPose demonstrated its capabilities even in scenarios with occlusions and non-frontal camera views [33].

OpenPose operates as a supervised convolutional neural network developed using the Caffe framework. Its strengths lie in the accuracy of facial, hand, and body pose detection, with resilience against interference from background environmental factors. A study by A. Viswakumar et al. highlighted OpenPose’s effectiveness in extreme lighting conditions, such as extremely bright and dimly lit environments, showcasing its robust key point estimation capabilities [34]. Moreover, OpenPose is versatile and applicable to single-person and multi-person pose recognition, exhibiting excellent recognition performance and high-speed processing.

When the system outputs an image, OpenPose undergoes initial image analysis through the first ten layers of the VGG-19 model to obtain a feature map. The image features are then input into two CNNs. The first CNN is used to predict the confidence map of human joints in the image, generating a separate confidence map for each joint. The second CNN combines the confidence map of detected human joints with the Part Affinity Field (PAF) to predict vectors representing the limbs of the human body [35].

In a study conducted by Q. Xu et al., OpenPose was utilized to extract skeletal diagrams of the human body during falls from internet videos and a dataset released by the University of Montreal in the fall. The trained model exhibited excellent recognition rates in conditions without obstructions and ample lighting. Conversely, recognition rates were lower in scenarios with excessive obstructions or low lighting [36].

Therefore, in this research, the intelligent fall posture analysis module relies on OpenPose’s posture detection, utilizing PAF technology for accurate and rapid detection of specific body postures in care recipients. This forms the basis for developing a UAV system that, upon detecting a care recipient through facial detection, can follow the care recipient, identify fall postures, categorize falls into different directions (front, back, left, right), record the temporal aspects of falls, assess fall severity, and notify medical personnel and family members of relevant fall information.

### 2.5. Teachable Machine

Teachable Machine is a no-code machine learning platform launched by Google. Even users with limited knowledge of programming concepts can easily train their machine-learning models on the platform’s website without writing any code. The process involves three training steps. First, users need to add classification content. Second, the Teachable Machine allows users to adjust the training parameters of the model to meet their specific needs. Even with limited expertise, users can train the model using its default training parameters. Finally, after training the model on the platform, users can test the model to confirm the training results. Currently, Teachable Machine offers training models for images, sounds, and human poses [37].

In this study, the Teachable Machine’s human pose training model was used to train models for four fall directions. These models were then compared with the intelligent tracking and fall detection algorithms developed in this study, serving as a benchmark for the recognition accuracy of falls in four different directions.

## 3. System Architecture and Module

In this study, the design and implementation of the Intelligent Dynamic UAV Tracking and Fall Detection (IDTFD) system for dynamic tracking and fall detection is divided into three steps.

**Figure 1:**
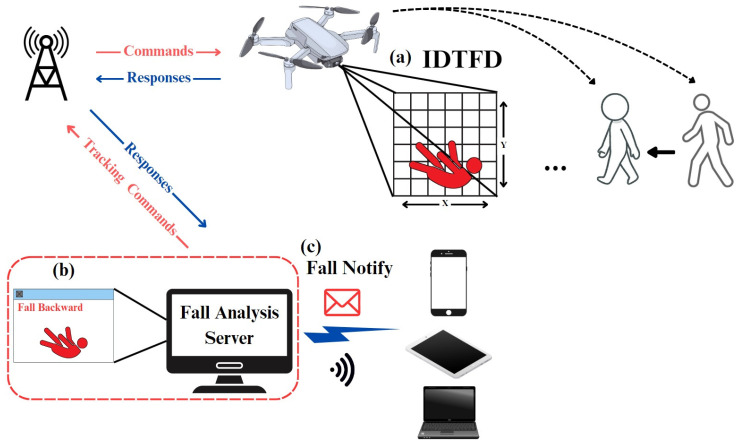
The overview diagram of dynamic tracking and fall detection using a drone. (**a**) Intelligent dynamic tracking and fall detection system. (**b**) Fall analsis server. (**c**) Fall notify.

The system remotely connects to the UAV, controlling its actions from takeoff to landing. The UAV then utilizes the SDTFD system to track the care recipient and detect falls. Initially, dynamic tracking and monitoring of the care recipient’s movements are performed using facial recognition technology that combines Dlib HOG plus Linear SVM with the UAV control module, ensuring that the care recipient remains within the UAV’s camera view. Next, OpenPose is employed to calculate the coordinates of the care recipient’s body joint points and segments in the image, enabling the system to recognize human postures. Finally, when the care recipient transitions from a normal posture to a fall posture, the study categorizes the fall into four different directions. This distinction is important as each fall direction may result in different types of bodily injuries. The UAV uses a fall classification model trained with OpenPose data to detect and identify the fall posture in these four directions and then transmits the information, as shown in Figure 1a.

Upon receiving the fall detection information from the UAV, the system displays the image captured by the UAV along with the name of the detected fall posture and simultaneously records the fall details and detection time. The fall information includes the severity of the fall, which is inferred from the duration the fall posture is maintained, estimating the potential bodily harm the care recipient may have suffered. This process is illustrated in Figure 1b. The system then sends the fall-related information detected by the UAV to pre-designated family members and medical personnel, ensuring that the care recipient receives timely medical treatment. This step is depicted in Figure 1c.

### 3.1. The Intelligent Tracking and Fall Detection Algorithm

This study leverages the open-source model OpenPose from Carnegie Mellon University (CMU), known for its accuracy in facial, hand, and body pose recognition. Combining this with Dlib’s facial recognition technology, and integrating it with drone control, tracking, and fall detection algorithms, we propose an intelligent tracking and fall detection system. Additionally, the study takes advantage of the agility of drones and the capabilities of deep learning algorithms with Convolutional Neural Network (CNN) approach to enhance the intelligent tracking and fall detection algorithm. This algorithm features automatic recognition, automatic assessment, dynamic adjustment of the drone’s camera direction and distance from the care recipient, as well as automatic tracking [38] and fall detection.

**Figure 2:**
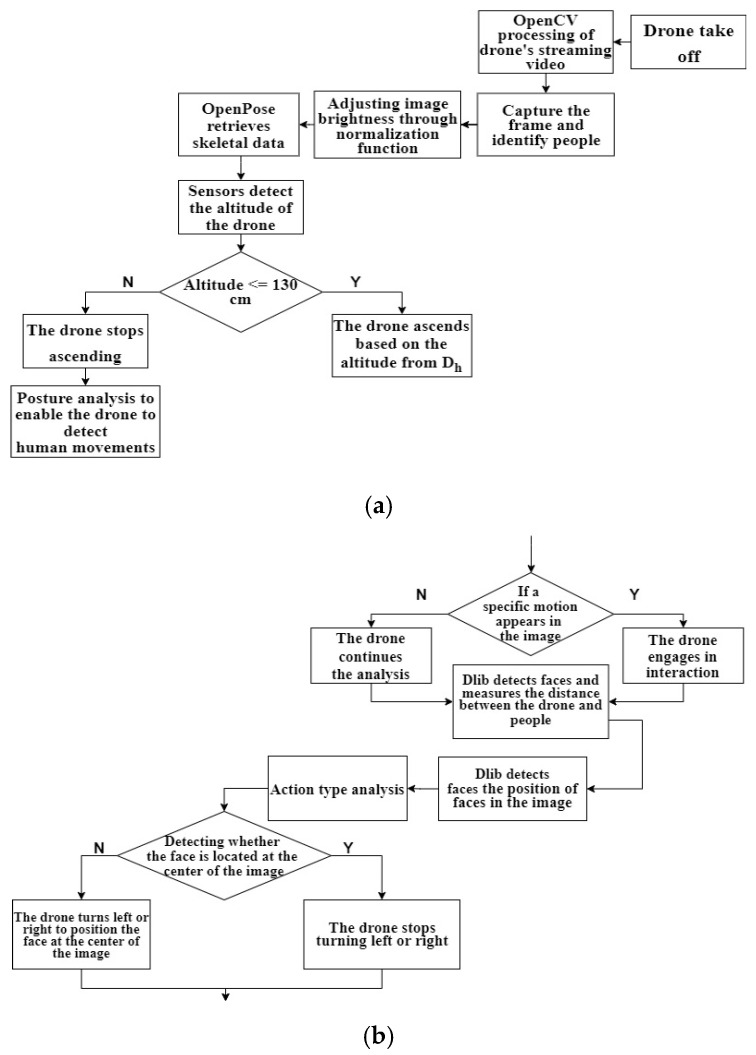

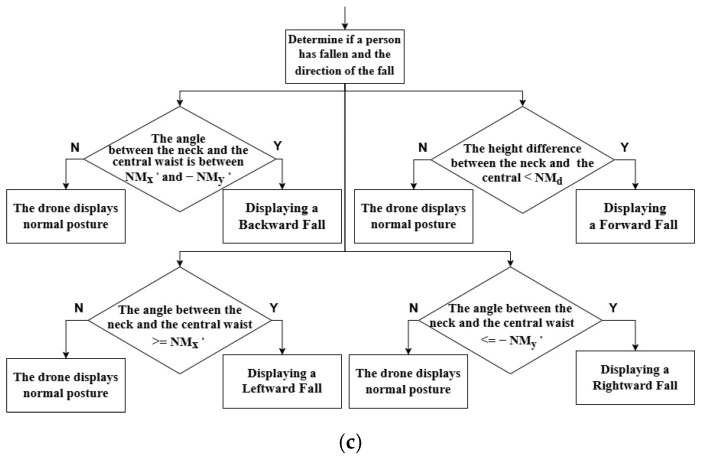
(**a**) Intelligent dynamic tracking and fall detection system: dynamic detection and recognition flowchart. (**b**) Intelligent dynamic tracking and fall detection system: real-time tracking and following flowchart. (**c**) Intelligent dynamic tracking and fall detection system: fall detection and analysis flowchart.

The proposed Intelligent UAV Dynamic Tracking and Fall Detection System, as illustrated in Figure 2, utilizes an intelligent fall detection algorithm and a pose estimation module, consisting of eight steps. Firstly, the system remotely connects to the UAV, automatically controlling takeoff from initiation to completion of preparation actions. Next, the system module captures images from the UAV using OpenCV and promptly sends them to a pre-set remote computer and mobile devices. In the third step, the system adjusts the image brightness using the standardization functions of OpenCV. The fourth step involves using the OpenPose model to label key points of the human body in the images, constructing the shape of the body.

In step five, the system employs Dlib HOG algorithm for facial detection to determine the care recipient’s distance and direction. The direction is crucial for adjusting the UAV’s camera orientation. In the sixth step, the system calculates the height differences and angles between the coordinates of OpenPose key points, classifying poses based on predefined criteria. In step seven, the UAV, through the ‘Intelligent Fall Posture Analysis Module’, determines if the care recipient has fallen and further identifies the direction of the fall.

Finally, if the system detects that the care recipient is in a predefined falling posture, it displays the abnormal pose on the remote screen. This allows the care recipient or their family to be informed and make judgments about the need for medical attention.

### 3.2. System Module and Functions

**Figure 3:**
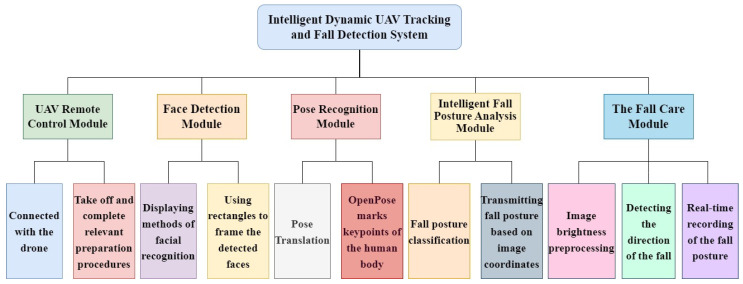
Functional decomposition diagram.

The ‘Intelligent Tracking and Fall Detection System’ in this paper consists of five modules, including the UAV remote control module, face detection module, pose recognition module, intelligent fall posture analysis module, and the fall care module. The functionalities under each module mainly involve image processing, face recognition, and fall posture classification, as illustrated in Figure 3.

#### 3.2.1. UAV Remote Control Module

Firstly, the system connects to the drone via a Wi-Fi network, after which the drone transmits flight data and altitude information. The drone then uses our proposed algorithm to intelligently track the care recipient and detect whether a fall posture is present. Meanwhile, the remote host displays the video feed from the drone at a predefined screen size. When the system detects a fall posture, it transmits relevant fall information to the care recipient’s designated family members or medical personnel. Additionally, the drone module communicates with the system in real time through the socket module.

#### 3.2.2. Face Detection Module

Furthermore, the intelligent dynamic drone system integrates the fast classification advantages of Dlib’s HOG and SVM techniques into this module. Dlib is employed to identify 68 facial landmarks, including eyebrows, eyes, nose, and mouth, using its pre-trained models. It leverages the HOG (Histogram of Oriented Gradients) method, capturing features of facial contours and distinguishing useful information from irrelevant details to simplify the image. Additionally, Dlib face detection moves through a predefined matrix, detecting facial features from the top left to the bottom right of the image, progressively excluding non-facial regions. Then, using a Linear SVM (Support Vector Machine), it classifies and identifies the facial region in the image.

The system utilizes deep learning for face detection and recognition, using Dlib’s facial recognition to determine the distance between the drone and the care recipient. This information is then used to adjust the drone’s flying distance from the care recipient. The Dlib HOG algorithm aids in determining the movement direction of the care recipient in the drone’s image, controlling the drone to sway left or right to keep the care recipient continuously in the frame. This achieves the goal of dynamic drone following of the care recipient.

**Figure 4:**
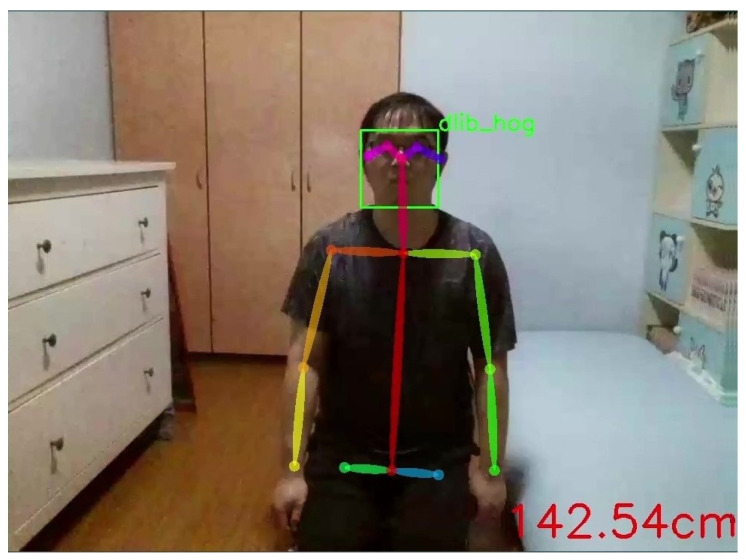
The green box represents the Dlib face recognition diagram.

After successful face detection by this method, Dlib outlines the face with a red box, as shown in Figure 4.

#### 3.2.3. Pose Recognition Module

This module is designed to perform posture analysis and segmentation algorithm programs for different human postures, enabling automatic analysis and judgment so that the drone can understand and interpret various human postures. Simultaneously, through this module, the drone can immediately notify the care recipient when they are in a fall posture.

**Figure 5:**
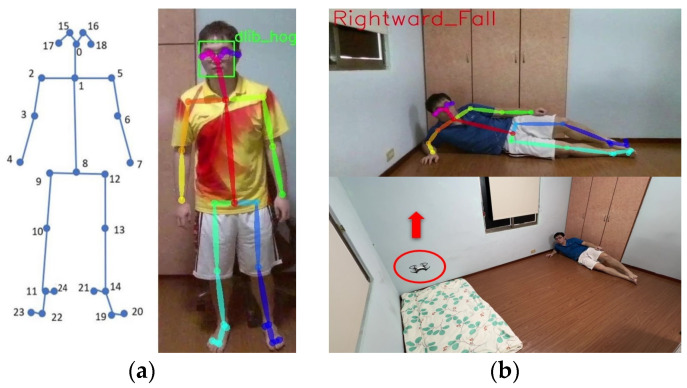
(**a**) OpenPose human pose estimation diagram; (**b**) OpenPose fall detection posture information display schematic.

The module identifies human postures based on the OpenPose model. OpenPose uses a CNN deep learning architecture to generate confidence maps for each joint position of the human body, and the Part Affinity Field (PAF) defined by OpenPose annotates the 2D vectors of each limb in the body. By integrating these two features, OpenPose can further predict each limb segment and successfully outline the entire body skeleton, consisting of 25 key points, as shown in Figure 5a. This study uses OpenPose to obtain the x and y coordinates of each joint point in the image. The fall classification model developed in this study then identifies whether the user is in a fall posture, allowing the drone to immediately notify the care recipient of the fall. Additionally, this function enables the host device to display the name of the fall posture, as shown in Figure 5b.

#### 3.2.4. Intelligent Fall Posture Analysis Module

The intelligent drone system in this study integrated the intelligent fall posture analysis algorithm to detects specific postures of care recipients resulting from falls using the fall classification model within the smart module. This allows the detection of four types of fall directions. Subsequently, the fall care module displays real-time fall information of the care recipient, recording the fall time and direction, thereby achieving the goal of providing care. In this study, the drone, through the correction feedback of the intelligent fall posture analysis algorithm, can instantly identify four types of fall postures: “Leftward fall”, “Rightward fall”, “Backward fall”, and “Forward fall”. The intelligent drone system interprets these fall postures to realize the purpose of drone-assisted care.

In the field of human action recognition, early methods mainly focused on using Convolutional Neural Networks (CNNs) to process the entire image [39,40]. While CNNs achieve high accuracy, they face significant challenges such as changes in lighting conditions in the images, high intra-class variance and low inter-class variance between similar actions, and cluttered backgrounds [41]. Moreover, CNNs analyze every pixel in the image, which makes them prone to detecting irrelevant elements such as the background or objects.

Currently, commonly used methods, such as OpenPose, extract human skeletal information from images [42,43,44]. The key advantage of these methods is their ability to extract human postures and use the skeletal data as input, allowing the Convolutional Neural Network to focus on important features such as posture and joint points rather than irrelevant features like background color and environmental factors. This approach not only significantly reduces computation time but also makes the model more lightweight while achieving high accuracy on test datasets.

Thus, this study employs OpenPose to extract 2D confidence maps of human joint points and Part Affinity Field (PAF) 2D vectors from images. The 2D confidence map generated by the model represents the detected joint points’ (x, y) coordinates and confidence level in the image, while the PAF predicts the unstructured pairwise relationships between human body parts (such as the wrist and elbow, or elbow and shoulder). The proposed “Intelligent Fall Posture Classifier” further utilizes the 2D confidence maps of human joint points and the PAF-predicted body part vectors to calculate the height difference between two joint points and the angle between the limb segment formed by the joint points and the vertical axis. These key feature values are then used as input for the Convolutional Neural Network (CNN) to classify fall postures. This approach not only allows the fall classification model trained in this study to gain the aforementioned advantages but also improves the classification model’s accuracy in identifying four different fall directions through the additional calculation of these key feature values.

**Figure 6:**
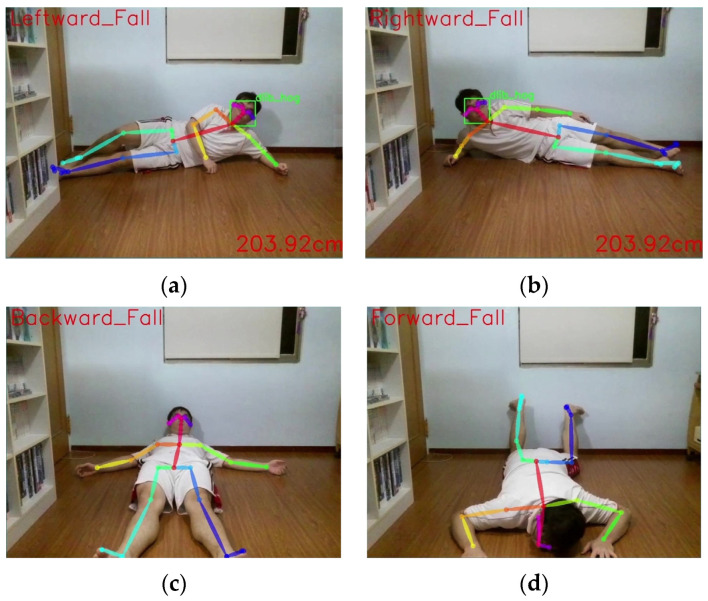
(**a**) Leftward fall; (**b**) rightward fall; (**c**) backward fall; (**d**) forward fall.

Moreover, by adjusting the weights assigned to these feature values in the feedback of the fall classification model, this study effectively reduces the loss function value and further enhances the model’s accuracy in recognizing fall postures in different directions. Ultimately, this system enables the drone to detect four different fall directions and notify pre-designated family members and healthcare professionals through the fall care module. The four specific fall postures are illustrated in Figure 6a–d below:

#### 3.2.5. The Fall Care Module

This mechanism, capable of recognizing the direction of falls, is the core module of the intelligent following and fall detection UAV in this study. The steps of the intelligent following and fall detection algorithm are as follows:

1. Drone Automatically Adjusts the Brightness of the Surrounding Image

According to the NORM_MINMAX formula in the normalization function of OpenCV, the module will calculate the percentiles of the image, remove outliers in pixel values, and scale the pixel values in the image to the specified percentile range. This enables automatic adjustment of the brightness in the image.

- Automated Adjustment of Image Brightness

The variables (i,j,x,y) in Equation (1) represent the x and y coordinates of any point in the image. The function src( ) refers to the source input array of the image, while the function dst( ) refers to the normalized and adjusted output array. The automated image brightness adjustment can be used by a drone to follow a care recipient and adjust the brightness when the surroundings are too dark or too bright, ensuring more stable fall detection. The following Equation (1) is used:

(1) $$\mathrm{dst}\left(i,j\right)=\frac{\left[\mathrm{src}\left(i,j\right)-\min\left(\mathrm{src}\left(x,y\right)\right)\right]\;\times \;(\max-\min)}{\max\left(\mathrm{src}\left(x,y\right)\right)-\min\left(\mathrm{src}\left(x,y\right)\right)}+\min$$

- Automated Adjustment of Ambient Brightness in Images

After the drone activates the camera, it immediately calculates the minimum and maximum values of the RGB channels’ percentiles in the image. Once these percentiles are obtained, the algorithm eliminates a small number of anomalous pixel values that fall outside this percentile range. Subsequently, the algorithm stretches the percentile value range to fit between the specified lower and upper limits. The range is not set between 0 and 255 to avoid potential pixel value overflow situations.

Through the normalization function using the NORM_MINMAX formula, the pixel values of the RGB channels are scaled to the specified percentile value range. This process enables the automatic adjustment of the image brightness around the drone.

In this study, the drone’s automatic following module is utilized. When the drone autonomously tracks a subject, it dynamically detects the brightness in the surrounding image. Consequently, it automatically adjusts the image to compensate for overly dark or bright backgrounds.

2. Detecting the Direction of the Care Recipient’s Fall

Once the human joints are identified by OpenPose, the drone immediately calculates and recognizes whether the posture of the care recipient in the image is a falling posture. If it is a falling posture, the system identifies the direction of the fall in the image as one of the four falling directions: “Leftward Fall”, “Rightward Fall”, “Backward Fall”, or “Forward Fall”. The drone continues to detect the direction of the fall in the image and records the duration during which the care recipient is identified as being in a falling posture.

- Detecting the Direction of the Fall

The variable $D_{t}$ in Equation (2) represents the angle between the line segment connecting two joint points and the vertical axis. To calculate $D_{t}$, the heights and widths of the two specified joint points in the image are subtracted to obtain their *x*-axis and *y*-axis coordinates. The angle is then calculated using the atan2() function from trigonometry, with π/2 subtracted from this value. Finally, the resulting angle is converted to degrees using the degrees() function to obtain $D_{t}$. The following Equation (2) is used:

(2) $$D_{t}=\mathrm{degrees}(\mathrm{atan}2\left(A_{h}-B_{h},A_{w}-B_{w}\right)-\frac{\mathrm{pi}}{2})$$

The variable $H_{D}$ in Equation (3) represents the height difference. To calculate $H_{D}$, the *y*-axis coordinates of the joint points obtained from OpenPose are used. The height difference is determined by subtracting the *y*-axis coordinates of the two joint points. The following Equation (3) is used:

(3) $$\mathrm{Two}\;\mathrm{joint}\;\mathrm{nodes}\;H_{D}=\mathrm{mid}\text{\_}\mathrm{Hip}[h]-\mathrm{neck}[h]$$

**Figure 7:**
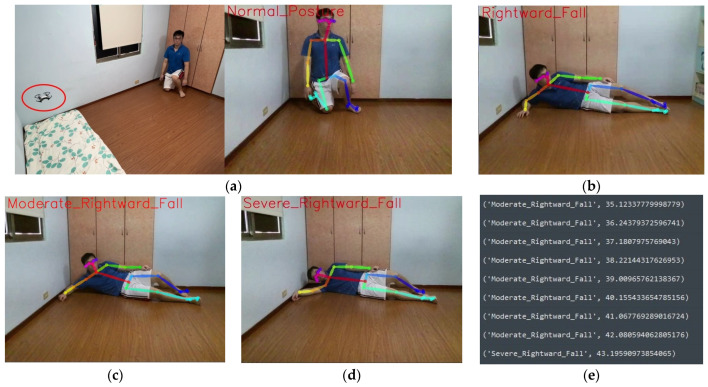
The drone detects when the care recipient falls and simultaneously records the duration of the fall. (**a**) Detected normal posture; (**b**) detected rightward fall; (**c**) displaying moderate rightward fall; (**d**) displaying severe rightward fall; (**e**) recorded information of the rightward fall.

Operation of the unmanned aerial vehicle fall detection and automatic follow-up care module can be divided into the following four steps between core modules:

Firstly, the “unmanned aerial vehicle” will inspect the captured environmental images to detect the presence of individuals. Therefore, individuals in the image will be recognized by OpenPose and depicted with body joints and lines, as shown in Figure 7a. Next, the joint points of the central hip and neck, as well as the key points of the neck and nose, are calculated to show the height difference in the image. Simultaneously, the line segment formed by the neck and nose, and the line segment formed by the left hip and right hip, are calculated to detect the direction of the fall, as shown in Figure 7b. Then, after detecting the falling posture, the drone will calculate how long the falling posture has been maintained. This is done by estimating the severity of the fall based on the length of time the fall lasted that the care recipient cannot get up due to the fall, as shown in Figure 7b–d. Therefore, through intelligent fall analysis, when this fall care module determines that the care recipient is in a fallen state, it further provides information on the duration of the fall to paramedics or doctors. This allows doctors to have clearer first-hand information about the fall when diagnosing the severity of the injury, making it an important reference for diagnosis.

Finally, the drone continues to follow the care recipient in the image, determining the direction of movement and whether there is a falling posture. The data are then sent back to the main system for the care recipient and their family to check, as shown in Figure 7d,e. The actual demonstration process of the entire unmanned aerial vehicle fall detection and automatic follow-up care calculation steps is depicted in the diagram. Figure 7a–e represent the detection of a rightward fall, identified as a severe rightward fall over a certain period, with the duration of the rightward fall recorded simultaneously.

3. Real-Time Recording of the Falling Posture

Next, while the drone follows the care recipient and detects whether there is a fall posture, it simultaneously transmits the care recipient’s posture and timestamp back to the main system. This allows the system to record in real time whether the care recipient has a fall posture. When a fall posture is detected, the system begins recording the direction of the fall and how long the fall posture lasts.

This study uses OpenPose as the method for human posture recognition, and the calculated height difference between joints and the angle between segments and the vertical axis are used as feature values, which are input into the CNN architecture for fall posture classification. The primary reason for this choice is due to its high accuracy and fast computation speed. For the same reasons, this study also uses Dlib for facial recognition. Dlib quickly captures facial features of the target using HOG and SVM techniques, which makes it characterized by fast computation speed and high accuracy.

## 4. Experiments and Results

This study focuses on the experiments involving drone-based facial recognition of care recipients and detection of various fall directions, as well as experiments designed for automatic brightness adjustment for forward falls. It simulates experimental results of recognition rates under various environments, as follows.

### 4.1. Experimental Environment

The experimental environment is divided into the following three parts.

#### 4.1.1. Detection of Care Recipient’s Facial Recognition

In this study, the functionality of the system’s facial detection module was tested using training models from OpenCV with Dlib or OpenCV with Haar. These models were employed to identify the care recipient’s facial features, enabling the drone to follow the care recipient. In the experimental phase, the method involved simulating the drone’s recognition of the care recipient’s face, ensuring continuous recognition of the facial features. The drone captured 100 consecutive photos using both Dlib and Haar models, and the accuracy of facial feature recognition by each method was assessed.

#### 4.1.2. The Environment for Identifying and Tracking the Direction of the Care Recipient’s Fall

This study utilized the PAF model, which was developed by OpenPose, as the training model. Further, it developed the identification of falling postures in various directions of the human body based on this model for application in drone detection. The experimental method of this study simulated four types of fall directions of the care recipient in various scenarios, allowing the drone to capture 100 consecutive photos. Subsequently, the module’s ability to correctly identify the four directions of falling was tested.

#### 4.1.3. Improving the Environment for Identifying Forward Falls of the Care Recipient

In this study, it was observed that when the drone detects the four directions of falling, OpenPose exhibits slight instability in identifying forward falls, where the head is downward. The feet are upward in the image. To address this issue, two additional conditions for judging forward falls were proposed. These conditions were simulated at a distance of 150 cm and in dim lighting conditions. The drone captured 100 consecutive photos, and then the effectiveness of the three criteria for judging forward falls was tested to improve the identification of forward falls of the care recipient. Furthermore, the interference of the four directions of falling in dim lighting conditions was simulated. Experiments were conducted both with and without brightness preprocessing to assess whether preprocessing methods could improve the identification of forward falls of the care recipient.

### 4.2. Experimental Design

Firstly, this study sought to test the ability of both Dlib and Haar to automatically recognize facial features on the smart drone. Lastly, based on different distances, indoor lighting conditions, and backgrounds, the study tests the smart drone’s ability to automatically detect “Leftward Fall”, “Rightward Fall”, “Backward Fall”, and “Forward Fall”. Therefore, the “Intelligent Dynamic Tracking and Fall Detection System” proposed in this study has three main functional modules, including the drone facial recognition function, the fall posture recognition and judgment function, and the system monitoring and recording function. We designed and conducted the following three sets of experiments, which were specifically assessed as follows:

(4) $$\mathrm{Accuracy}\;=\frac{\mathrm{Number}\;\mathrm{of}\;\mathrm{Correct}\;\mathrm{Instances}}{\mathrm{Total}\;\mathrm{Number}\;\mathrm{of}\;\mathrm{Instances}\;\mathrm{Tested}}$$

#### 4.2.1. The Recognition Rates of Facial Detection Using Dlib and Haar

**Figure 8:**
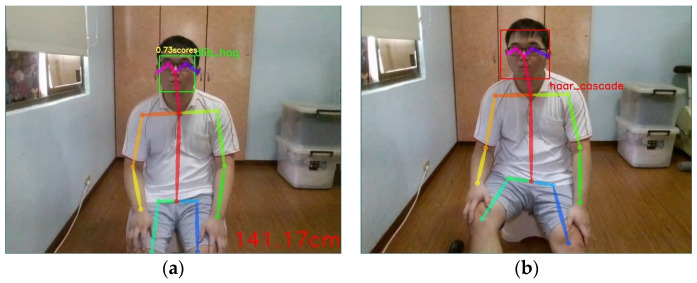
The drone utilizes Dlib hog and Haar feature detection to recognize the care recipient’s face. (**a**) Dlib HOG face recognition; (**b**) Haar feature-based face recognition.

This experiment consisted of two parts: Dlib and Haar, each conducting facial feature detection experiments. When the drone successfully detects a face, it displays a green (Dlib) or red (Haar) box around the face. Considering the drone’s perspective towards the care recipient, this experiment focuses on frontal face detection (See Figure 8). Each facial recognition method was tested 100 times. The results are shown in Table 1 below.

In this experiment, during the frontal face detection of the care recipient by the drone, the facial recognition rates were 99% (OpenCV + Dlib) and 93% (OpenCV + Haar), respectively. The reason for the failure in facial recognition for both methods was when the care recipient tilted their head. However, during the experiment, Dlib experienced only a momentary failure in facial recognition. Even when the care recipient’s head was tilted, Dlib managed to successfully recognize the face. On the other hand, Haar’s facial recognition failed whenever the care recipient tilted their head. This could be attributed to the Haar facial recognition model not being trained on facial features when the head is tilted, resulting in unrecognized faces.

#### 4.2.2. Detection and Recognition Rates of Four Falling Directions at Different Distances (Near, Far)

**Figure 9:**
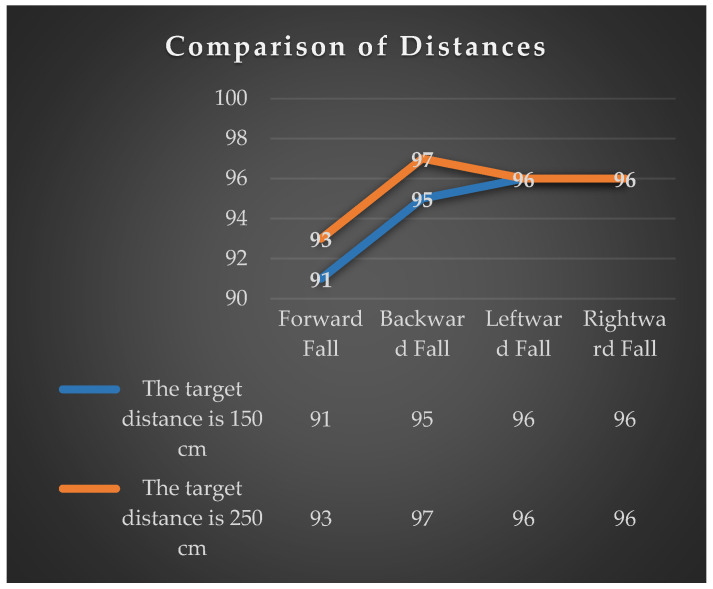
The accuracy of fall detection and recognition at close and far distances.

This experiment was divided into two categories: short-distance (150 cm) and long-distance (250 cm), each conducting detection experiments on the care recipient’s transition from normal to falling posture. When the drone correctly displays the name of the detected fall posture, it is considered a successful detection. The experiment is categorized into four falling directions faced by the drone, with each direction undergoing 100 experiments. The experimental results are shown in Figure 9.

In this experiment, when the drone conducted experiments to detect “Leftward Fall”, “Rightward Fall”, “Backward Fall”, and “Forward Fall” of the care recipient, the overall detection rates for common falls remained above 91%. In fact, during experiments for “Leftward Fall”, “Rightward Fall”, and “Backward Fall”, detection rates surpassed 95% at both 150 cm and 250 cm distances. However, in the experiments for detecting “Forward Fall”, the recognition accuracy was comparatively lower, at 91% (150 cm) and 93% (250 cm). The reason for detection errors in “Forward Fall” scenarios is attributed to the presence of shadows on the face of the subject due to overhead lighting, resulting in a darker appearance in the image. Consequently, OpenPose often loses key points above the neck when detecting, leading to recognition failures. For the other three types of falling postures, detection failures occur because the subject does not reach the angle criteria for determining these fall postures during the transition from normal to falling postures.

#### 4.2.3. The Detection and Recognition Rates of Four Types of Falls in Different Lighting Environments

**Figure 10:**
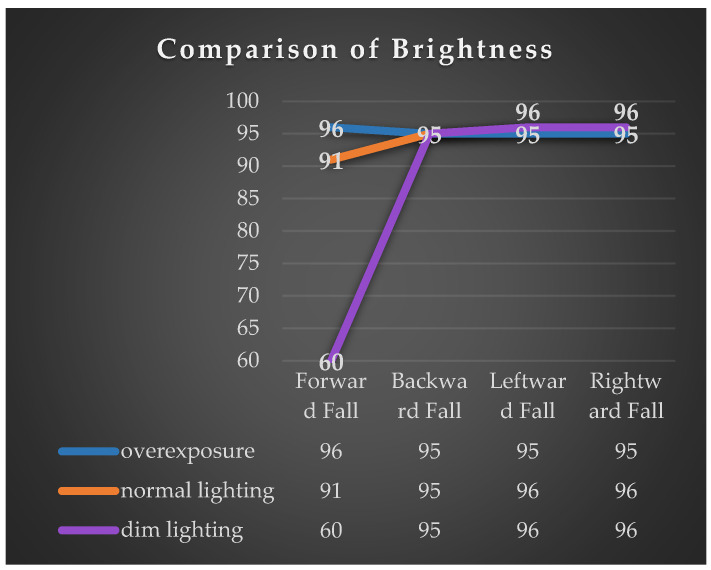
Accuracy of fall detection recognition in different lighting environments.

**Figure 11:**
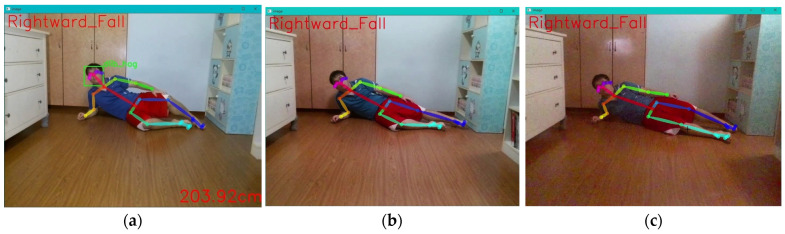
Testing conditions in different lighting environments. (**a**) Overexposure; (**b**) normal lighting; (**c**) dim lighting.

This experiment tested the detection accuracy of four types of falls captured by drones in different lighting environments. The experiment involved adjusting the lighting conditions in a closed room to simulate overexposure, normal lighting, and dim lighting, as depicted in the illustration. The accuracy of the detection of the four types of fall directions by the drone was evaluated through these three lighting environments. Each type of fall direction was tested 100 times under continuous shooting conditions. The results of the experiment indicate that the recognition rates of leftward, rightward, and backward falls remain above 95% in different lighting conditions, as shown in Figure 10. However, for forward falls, it was observed that as the lighting decreased, the success rate of recognition also decreased. This is primarily because the criteria for identifying forward falls are based on the line segment from the neck to the nose. When the facial area in the image becomes darker, OpenPose tends to lose key points above the neck, leading to recognition failure. See Figure 11.

#### 4.2.4. Recognition Rate of Fall Detection in Four Directions Under Different Background Environments Captured

**Figure 12:**
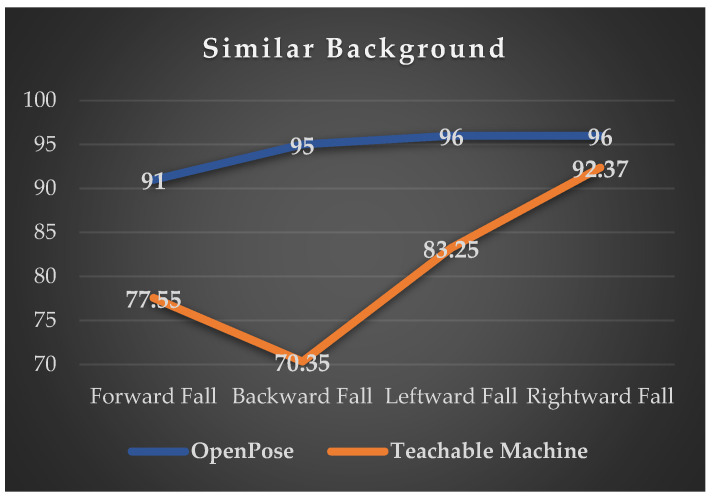
Accuracy of fall detection recognition under similar backgrounds.

**Figure 13:**
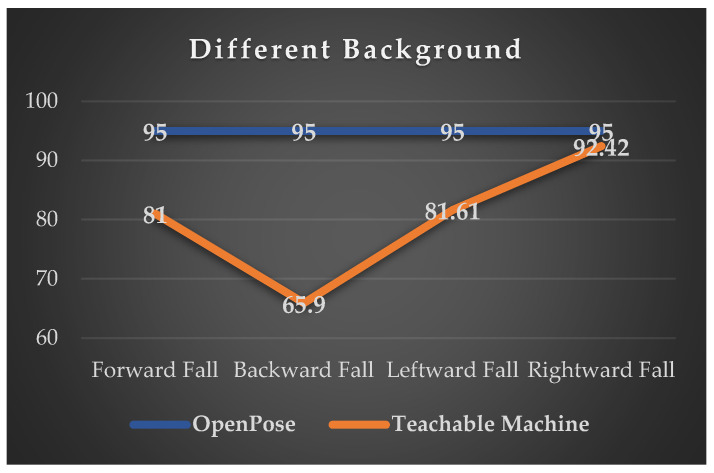
Accuracy of fall detection recognition under different backgrounds.

In this experiment, we investigated the impact of changing backgrounds on the accuracy of identification by the system developed based on OpenPose and the Pose Project training model of Google Teachable Machine for the recognition rates of four falling directions. The experimental results are shown in Figure 12 and Figure 13.

In both similar and dissimilar background scenarios, the recognition accuracy of the four fall directions detected by the fall detection system developed based on OpenPose in this study, compared to the Pose Project training model of the Google Teachable Machine system, is generally above 92%, except for the rightward fall direction which exhibits similar recognition accuracy. However, for the other three fall directions, the recognition accuracy is slightly lower compared to that of the system developed in this study. The main reason lies in the similarity between the Teachable Machine system and OpenPose detection methods, facilitated by the Pose Project training model, making it less susceptible to changes in surrounding backgrounds, hence resulting in fewer recognition failures. Nonetheless, experimental results indicate that while the Teachable Machine is capable of recognizing fall postures, it tends to confuse directional falls, leading to instances where a subject displaying a forward fall posture might be incorrectly identified as leftward, rightward, or backward falls. Additionally, among the four fall postures, the recognition accuracy for rear falls is the lowest. This can be attributed to the inability of the Teachable Machine to detect facial key points in certain rear fall postures, resulting in misidentification as one of the other three fall directions.

#### 4.2.5. Improvement of Forward Fall Detection Recognition Rate in Dimly Lit Environments

In this experiment, based on the previous experiments conducted in different lighting conditions for four types of fall directions, we observed that in dim lighting conditions, the forward fall was affected by shadowing on the head, resulting in key point loss above the neck by OpenPose and subsequently leading to a recognition rate of only 60%. Therefore, we tested whether adjusting the brightness through the normalization function (NORM_MINMAX) in OpenCV could improve the recognition rate of forward falls in dim lighting conditions. The experimental results are shown in Table 2 below.

**Figure 14:**
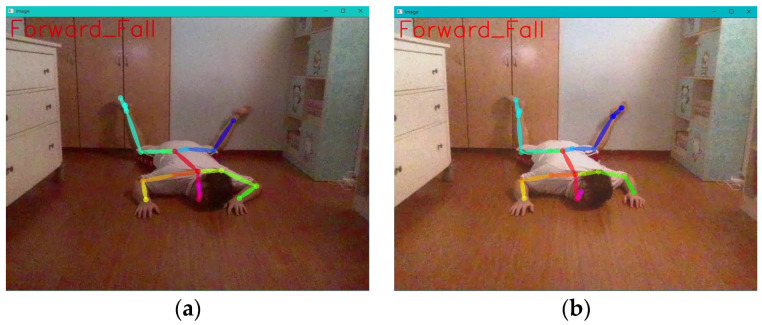
Testing scenarios in environments with dim lighting and automated adjustment of image brightness. (**a**) Dim lighting; (**b**) automated adjustment of image brightness.

In this experiment, we observed that after undergoing automatic adjustment of image brightness, the recognition rate of forward falls increased from the original 60% to 93%. Additionally, based on Figure 14 below, we can clearly see that the part of the forward fall affected by shadowing has been improved. This improvement enables OpenPose to detect key points above the neck more accurately, increasing the recognition rate.

#### 4.2.6. The Improvement of Forward Falls

The comparison between the intelligent fall posture analysis module in this study and the Google Teachable Machine’s Pose Project training model for forward fall detection shows that our system achieves a 91% accuracy rate for forward fall recognition, slightly higher than the 77.55% accuracy rate of the Teachable Machine system. However, it was observed that forward falls in our system are prone to interference from head shadow obstructions, which can cause OpenPose to fail in recognizing postures, and this issue becomes more severe in dimly lit environments, as shown in Table 2.

Additionally, during forward fall detection, there is a tendency for joint occlusion to occur. To address this, the experiment adjusted the conditions for forward fall judgment. Under the condition where the drone was positioned 150 cm away from the care recipient, the system conducted 100 consecutive tests. The experimental results are shown in Table 3.

In this experiment, we first improved the detection of forward falls by modifying the I_OPose method, which detects the height difference between the neck and nose, and replaced it with the E_HN_OPose method, which detects the height difference between the waist and neck. This change led to an increase in forward fall recognition accuracy to 93%. The main reason for this improvement is that the drone’s view of the back is less affected by shadows, allowing OpenPose to more clearly identify key points of the posture during forward fall detection.

Furthermore, we discovered that when using E_HN_OPose to detect the process of the care recipient transitioning from standing to falling, the height difference did not drop below the threshold, causing the drone to fail to detect the fall. Additionally, during forward fall detection, body parts would sometimes occlude each other, leading to missing or incorrectly predicted joint points.

**Figure 15:**
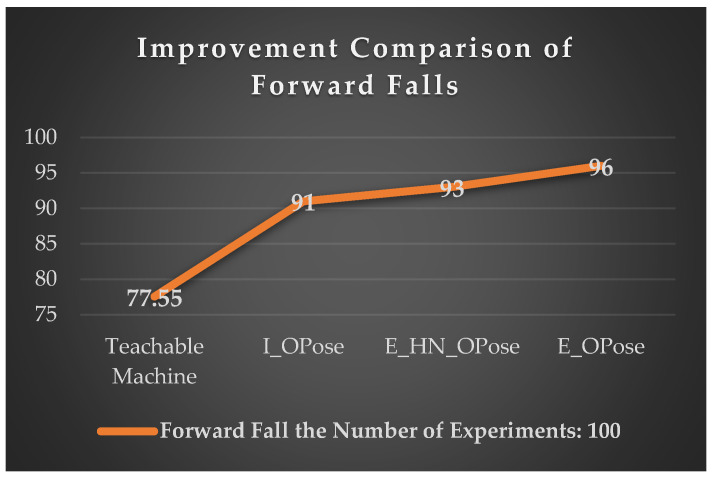
Recognition accuracy of fall detection with different forward fall detection methods.

To address these issues, we adopted the relative positions between joints by using the E_OPose method to detect either the height difference between the waist and neck or between the neck and nose. This approach combines the advantages of I_OPose and E_HN_OPose, allowing the two methods to complement each other and enhancing recognition by using these key features. The experiment results show that the recognition success rate increased from 93% to 96%. As a result, the overall recognition accuracy of the experiment significantly improved from 77% to 96%, as seen in Figure 15.

## 5. Discussion

This experiment mainly consists of three parts: the system’s recognition of the four fall directions of the care recipient in terms of detection accuracy at different distances, different lighting conditions, and different backgrounds; the system’s improvement of forward fall detection in dim lighting environments; and the system’s method of improving forward fall detection by modifying judgment criteria. The experiment demonstrates that the accuracy of the intelligent UAV dynamic tracking and fall detection system in both facial recognition and improving the detection of the four fall directions approaches 95%.

### 5.1. In-Depth Discussion on the Algorithm and Experimental Results for Forward Fall Detection

In the intelligent fall posture analysis module developed in this study, fall detection across four directions—forward, leftward, rightward, and backward—was tested under the same conditions, with the drone positioned 150 cm away. It was observed that forward fall detection had a slightly lower accuracy of 91%, compared to the other directions. The forward fall detection using I_OPose focuses on the height difference between the neck and nose key points. During experiments, we found that one of the main reasons for the lower accuracy was that the care recipient’s face, when in a forward fall posture, was often obscured by light and shadows, causing the facial key points to be lost and resulting in OpenPose failing to recognize the posture.

To address this issue, we changed the detection method to focus on the back of the body. The E_HN_OPose method detects the height difference between the waist and neck key points, effectively reducing the problem of facial key point loss caused by head shadow occlusion in I_OPose. This adjustment increased the drone’s detection accuracy from 91% to 93%. However, E_HN_OPose still encountered issues when detecting the process of the care recipient falling or during forward falls, as the height difference sometimes did not meet the detection threshold. Additionally, joint occlusion during the fall caused OpenPose to misidentify or lose key points, resulting in detection failures.

To resolve these challenges, we proposed the E_OPose method, which detects either the waist-to-neck or neck-to-nose height difference. By combining the advantages of both I_OPose and E_HN_OPose, we achieved a complementary effect. This approach mitigated the problem of I_OPose failing due to head shadow occlusion, and also resolved the issues of E_HN_OPose struggling to detect the falling process and joint occlusion causing key point loss or incorrect predictions. The experimental results of this study confirmed that the detection accuracy improved from 93% to 96% when using this combined approach with the drone.

### 5.2. Discussion on Improving Openpose in Intelligent Dynamic Tracking and Fall Detection System

Furthermore, in dimly lit environments, the intelligent dynamic tracking and fall detection system addresses the issue of certain areas being too dark during drone detection of care recipients by implementing automatic adjustment of image brightness. This enhancement aims to mitigate the potential loss of key points by OpenPose due to poorly illuminated regions. As indicated by the experimental results in Table 2, we observed a significant improvement in the detection accuracy of forward falls from 60% to 93% after implementing automatic adjustment of image brightness. Additionally, our research system provides a timeline for recording fall events and their severity. Upon detecting a fall posture in the care recipient, the system begins recording the direction of the fall and the duration of maintaining the fallen posture. Leveraging the recorded fall timeline, we propose a method to estimate the severity of falls by calculating the duration of maintaining the same fall posture. These functionalities effectively address the limitations of OpenPose in recording fall posture duration and assessing fall severity, enabling care recipients and their families to be informed about the severity of detected falls for prompt medical attention.

### 5.3. Intelligent Tracking and Fall Detection Algorithm vs. Google Teachable Machine

The intelligent tracking and fall detection algorithm of this study was compared with Google Teachable Machine’s Pose Project training model, as shown in Figure 12 and Figure 13. In both similar and dissimilar background conditions, the accuracy of the intelligent tracking and fall detection algorithm for detecting and recognizing the four directions of fall posture was higher than that of the Google Teachable Machine. Particularly, the accuracy of identifying the backward fall posture saw the greatest improvement, with recognition accuracy increasing from 70.35% to 95% in similar background conditions and from 65.9% to 95% in dissimilar background conditions.

The reason behind this lies in the fact that our system algorithm utilizes methods based on OpenPose and PAF, combined with the intelligent tracking and fall detection algorithm proposed in this study. As a result, both algorithms can extract joint points from various body parts during posture recognition. However, in experiments, it was found that the Pose Project training model provided by the Google Teachable Machine platform tends to lose joint points, leading to lower accuracy rates in both experiments. Except for the accuracy rate of identifying the rightward fall posture, which reaches 92%, the accuracy rates for the other three directions of fall posture range between 65.9% and 83.25%. In contrast, the fall detection algorithm proposed in this study can achieve accuracy rates of at least 90% to 95% for all four directions of falls.

Furthermore, compared to the Google Teachable Machine system, our algorithm calculates the height difference between two joint points or the angle formed by a line segment connecting two key points and the vertical axis, bringing about its high variability. This characteristic allows for higher accuracy and variability in detecting specific posture values, making it possible to achieve higher accuracy rates with greater variability.

## 6. Conclusions

The unmanned aerial vehicle (UAV) intelligent tracking and fall detection system proposed in this paper combines Dlib face detection with OpenPose deep learning algorithms. Developed using Python libraries, the system continuously records the timing of falls and estimates the severity of falls based on their duration. This enables the system to autonomously track and provide immediate fall care, addressing the limitations of fixed-point monitors unable to track care recipients. In aging societies, where elderly individuals may experience falls without timely assistance, the inability to accurately detect the direction and severity of falls complicates the transmission of fall information to medical professionals, potentially delaying necessary treatment.

The system promptly detects and analyzes faces and postures, determining whether the UAV has a direct view of the care recipient. Then, using the fall care module, it logs the timestamp based on whether the recipient exhibits a falling posture and the fall’s direction. When a fall is confirmed, the system estimates the fall’s severity based on its duration. The experimental results confirm that the system developed in this study can accurately and promptly identify falls, providing effective continuous detection and tracking with mobility capabilities. This system significantly improves the monitoring range and efficiency compared to traditional stationary cameras. Moreover, during drone operation, the system can determine the fall direction based on the care recipient’s posture and simultaneously record details such as the fall direction, timestamp, severity, and real-time images.

This not only overcomes the limitations of blind spots in traditional surveillance systems but also provides detailed fall detection information that helps medical professionals accurately identify appropriate X-ray examination areas. This detailed fall data supports subsequent diagnosis and treatment, ensuring timely and targeted medical interventions. The system effectively addresses the growing caregiver shortage in an aging society and enhances the utilization of limited healthcare resources, allowing them to be used more efficiently.

In the research, it was observed that OpenPose encounters issues with joint occlusion when detecting fall postures in all four directions. Specifically, during the detection of forward falls, the lower body below the waist often suffers from missing joint points. The primary reason for this is that when the care recipient remains in a forward fall posture, their legs tend to compress, causing joints to overlap, making it difficult for OpenPose to correctly identify joints. Consequently, it may result in misinterpretations or failure to detect the posture.

To address these issues, which negatively impact the system’s fall detection accuracy, this study proposes an “intelligent fall posture analysis algorithm”. By utilizing joint coordinates and calculating both the height differences between joints and the angles between body segments and the vertical axis, the system accurately analyzes the parameters for fall postures in all four directions. These parameters are crucial features for fall classification and help establish a robust fall classification model. As a result, the system achieves real-time detection with a high accuracy rate exceeding 95% across all four directions in fall posture detection tests. Specifically, the detection accuracy for forward, backward, leftward, and rightward falls are improved to reached 96%, 95%, 96%, and 96% respectively.

Moreover, during the automated brightness adjustment experiments, we found that, under certain conditions, image noise could blur the image. This, in turn, destabilizes OpenPose’s ability to reliably detect body parts, further highlighting the need for enhanced detection algorithms and improvements with the pre-processing effect of insufficient image brightness in this study. Therefore, this study provides the investigating methods to utilize image sharpness and noise reduction processing which may help reduce image noise, thereby enhancing the stability of OpenPose in detecting human body postures under automatic brightness adjustment conditions.

In future research, we will consider further improvements that can be made by modifying the OpenPose training model to address issues where specific poses cause significant body part overlap, leading to compressed images that result in OpenPose losing key points in the overlapped regions. In addition, detecting a sequence of movements associated with falls could help identify hazardous poses and assess the severity of falls among the elderly based on the duration of these postures. This would allow the system to provide healthcare professionals with timely and accurate fall-related information for further medical evaluation. Strengthening this system to deliver precise fall data to medical personnel could support subsequent treatment and necessary care for patients, offering valuable assistance.

## Figures and Tables

**Table 1:** Experimental results of face detection using Dlib and Haar.

System Comparison	The Target Distance Is 150 cm
OpenCV + Dlib	99%
OpenCV + Haar	93%
Number of Experiments	100

**Table 2:** Improvement of dim background in forward fall detection recognition experiment results.

Comparison of Brightness Adjustments	Forward Fall
Dim Lighting	60%
Automated Adjustment of Image Brightness	93%
Number of Experiments	100

**Table 3:** The experimental results of fall detection recognition using different methods for identifying forward falls.

Comparison of Different Methods	Forward Fall
Teachable Machine	77.55%
I_Opose (The height difference from the neck to the nose)	91%
E_HN_Opose (The height difference between the waist and the neck)	93%
E_Opose (The height difference between the waist and the neck or the height difference between the neck and the nose)	96%
Number of Experiments	100

## Data Availability

Data are contained within the article.

## References

[1] Yeh M.-J. (2020). Long-term care system in Taiwan: The 2017 major reform and its challenges. Ageing Soc..

[2] Huang Y.-F., Lin C.-B., Dong Z., Kuan W.-K. (2021). A Framework for Fall Detection Based on Openpose Skeleton And Lstmgru Models. Appl. Sci..

[3] Chen W., Jiang Z., Guo H., Ni X. (2020). Fall Detection based on key points of human-skeleton using openpose. Appl. Sci..

[4] Huang Z., Liu Y., Fang Y., Horn B.K.P. Video-based Fall Detection for Seniors with Human Pose Estimation. Proceedings of the 2018 4th International Conference on Universal Village (UV).

[5] Lapierre N., Neubauer N., Miguel-Cruz A., Rincon A.R., Liu L., Rousseau J. (2018). The state of knowledge on technologies and their use for fall detection A scoping review. Int. J. Med. Inform..

[6] World Health Organization (WHO) Falls. https://www.who.int/news-room/fact-sheets/detail/falls.

[7] Kannus P., Sievänen H., Palvanen M., Järvinen T., Parkkari J. (2005). Prevention of falls and consequent injuries in elderly people. Lancet.

[8] Leung W.-S., Chi H.-T., Hu M.-H., Lin M.-T. (2005). Fall Mechanism and Injury Severity in Community-Dwelling Older People. Formos. J. Phys. Ther..

[9] Tolkiehn M., Atallah L., Lo B., Yang G.Z. Direction sensitive fall detection using a triaxial accelerometer and a barometric pressure sensor. Proceedings of the 2011 Annual International Conference of the IEEE Engineering in Medicine and Biology Society.

[10] Christiansen T.L., Lipsitz S., Scanlan M., Yu S.P., Lindros M.E., Leung W.Y., Adelman J., Bates D.W., Dykes P.C. (2020). Patient Activation Related to Fall Prevention A Multisite Study. Jt. Comm. J. Qual. Patient Saf..

[11] Chen J., Kwong K., Chang D., Luk J., Bajcsy R. Wearable sensors for reliable fall detection. Proceedings of the 2005 IEEE Engineering in Medicine and Biology 27th Annual Conference.

[12] Karantonis D.M., Narayanan M.R., Mathie M., Lovell N.H., Celler B.G. (2006). Implementation of a real-time human movement classifier using a triaxial accelerometer for ambulatory monitoring. IEEE Trans. Inf. Technol. Biomed..

[13] Korats G., Hofmanis J., Skorodumovs A., Avots E. Fall detection algorithm in energy efficient multistate sensor system. Proceedings of the 2015 37th Annual International Conference of the IEEE Engineering in Medicine and Biology Society (EMBC).

[14] Sim S.Y., Jeon H.S., Chung G.S., Kim S.K., Kwon S.J., Lee W.K., Park K.S. Fall detection algorithm for the elderly using acceleration sensors on the shoes. Proceedings of the 2011 Annual International Conference of the IEEE Engineering in Medicine and Biology Society.

[15] Disability-Adjusted Life Years (DALYs). https://www.who.int/data/gho/indicator-metadata-registry/imr-details/158.

[16] Wang S., Chen L., Zhou Z., Sun X., Dong J. (2016). Human fall detection in surveillance video based on PCANet. Multimed. Tools Appl..

[17] Igual R., Medrano C., Plaza I. (2013). Challenges, issues and trends in fall detection systems. BioMed. Eng. Online.

[18] Pannurat N., Thiemjarus S., Nantajeewarawat E. (2014). Automatic fall monitoring: A review. Sensors.

[19] Chahed S., Marcon E., Sahin E., Feillet D., Dallery Y. (2009). Exploring new operational research opportunities within the Home Care context: The chemotherapy at home. Health Care Manag. Sci..

[20] Chiang C.-Y., Chen Y.-L., Yu C.-W., Yuan S.-M., Hong Z.-W. An Efficient Component-Based Framework for Intelligent Home-Care System Design with Video and Physiological Monitoring Machineries. Proceedings of the 2011 Fifth International Conference on Genetic and Evolutionary Computing (ICGEC).

[21] Estudillo-Valderrama M.A., Roa L.M., Reina-Tosina J., Naranjo-Hernandez D. (2009). Design and Implementation of a Distributed Fall Detection System–Personal Server. IEEE Trans. Inf. Technol. Biomed..

[22] Juang L.-H., Wu M.-N. (2015). Fall Down Detection Under Smart Home System. J. Med. Syst..

[23] Kim B. (2023). Implementation of Falling Accident Monitoring and Prediction System using Real-time Integrated Sensing Data. KSII Trans. Internet Inf. Syst..

[24] Lecun Y., Bengio Y., Hinton G. (2015). Deep learning. Nature.

[25] Mubashir M., Shao L., Seed L. (2013). A survey on fall detection: Principles and approaches. Neurocomputing.

[26] Nandi P., Anupama K.R., Agarwal H., Patel K., Bang V., Bharat M., Guru M.V. (2024). Inertial measurement and heart-rate sensor-based dataset for geriatric fall detection using custom built wrist-worn device. Data Brief..

[27] Bourke A.K., Lyons G.M. (2008). A threshold-based fall-detection algorithm using a bi-axial gyroscope sensor. Med. Eng. Phys..

[28] Micucci D., Mobilio M., Napoletano P. (2017). UniMiB SHAR: A dataset for human activity recognition using acceleration data from smartphones. Appl. Sci..

[29] Wang Y., Wu K., Ni L.M. (2016). WiFall: Device-Free Fall Detection by Wireless Networks. IEEE Trans. Mob. Comput..

[30] Wafiq M.F., Taz M., Nowrin F., Chowdhury A.M. An IoT-Based Bed Fall Prediction System Using Force Sensitive Resistor. Proceedings of the 2023 IEEE Region 10 Symposium (TENSYMP).

[31] Nadee C., Chamnongthai K. Ultrasonic array sensors for monitoring of human fall detection. Proceedings of the 2015 12th International Conference on Electrical Engineering/Electronics, Computer, Telecommunications and Information Technology (ECTI-CON).

[32] Santhoshi P.M., Thirugnanam M. (2016). A novel framework for fall detection by using ambient sensors and voice recording. Res. J. Pharm. Biol. Chem. Sci..

[33] Kim W., Sung J., Saakes D., Huang C., Xiong S. (2021). Ergonomic postural assessment using a new open-source human pose estimation technology (OpenPose). Int. J. Ind. Ergon..

[34] Viswakumar A., Rajagopalan V., Ray T., Parimi C. Human Gait Analysis Using OpenPose. Proceedings of the 2019 Fifth International Conference on Image Information Processing (ICIIP).

[35] Cao Z., Hidalgo G., Simon T., Wei S.-E., Sheikh Y. OpenPose: Realtime Multi-Person 2D Pose Estimation Using Part Affinity Fields. Proceedings of the IEEE Conference on Computer Vision and Pattern Recognition (CVPR).

[36] Xu Q., Huang G., Yu M., Guo Y. (2019). Fall prediction based on key points of human bones. Phys. A Stat. Mech. Appl..

[37] Google Teachable Machine FAQ. https://teachablemachine.withgoogle.com/faq.

[38] Yao C.-B., Kao C.-Y., Lin J.-T. (2023). Drone for Dynamic Monitoring and Tracking with Intelligent Image Analysis. Int. J. Intell. Autom. Soft Comput..

[39] Garg S., Saxena A., Gupta R. (2023). Yoga pose classification: A cnn and mediapipe inspired deep learning approach for real-world application. J. Amb. Intel. Hum. Comp..

[40] Cheron G., Laptev I., Schmid C. P-CNN: Pose-based cnn features for action recognition. Proceedings of the 2015 IEEE International Conference on Computer Vision (ICCV).

[41] Girish D., Singh V., Ralescu A. Understanding action recognition in still images. Proceedings of the 2020 IEEE/CVF Conference on Computer Vision and Pattern Recognition Workshops (CVPRW).

[42] Lin H., Tse R., Tang S.-K., Chen Y., Ke W., Pau G. Near-realtime face mask wearing recognition based on deep learning. Proceedings of the 2021 IEEE 18th Annual Consumer Communications & Networking Conference (CCNC).

[43] Rong F., Tongtong W., Zuying L., Fuqing D., Xuejun Q., Ping G. Learning behavior analysis in classroom based on deep learning. Proceedings of the 2019 Tenth International Conference on Intelligent Control and Information Processing (ICICIP).

[44] Fazil R., Binali D.S., Sasmini A., Shakeel N., Lakmal R., Chethana L. Infinity yoga tutor: Yoga posture detection and correction system. Proceedings of the 2020 5th International Conference on Information Technology Research (ICITR).

